# Effects of Partial Replacement of Soybean Meal with Defatted *Hermetia illucens* Meal in the Diet of Laying Hens on Performance, Dietary Egg Quality, and Serum Biochemical and Redox Indices

**DOI:** 10.3390/ani13030527

**Published:** 2023-02-02

**Authors:** Patrycja Zawisza, Beata Szymczyk, Anna Arczewska-Włosek, Kinga Szczepanik

**Affiliations:** Department of Animal Nutrition and Feed Science, National Research Institute of Animal Production, 32-083 Balice, Poland

**Keywords:** *Hermetia illucens* meal, laying performance, egg composition, fatty acids

## Abstract

**Simple Summary:**

The search for a substitute for soybean meal (SBM) as a feed source in compound feeds has been a significant challenge in the poultry industry. Substitutes with a similar nutritional value and from local sources are desirable. Thanks to changes in European Union law, insects can be used in poultry feed since 2021. This enables the usage of meal from the insect *Hermetia illucens*, which has been thoroughly tested for usefulness as feed. This study aimed to determine whether the partial replacement of SBM with defatted *H. illucens* larvae meal in the diet of laying hens is possible, without a negative impact on performance indices, egg quality, and health.

**Abstract:**

This study was carried out on 96 caged Bovans Brown laying hens at an initial age of 25 weeks, which were randomly assigned to four experimental groups of 12 replicates (cages) of two hens per cage. The control group hens received a diet containing 20% soybean meal (SBM), without *Hermetia illucens* larvae meal (HIM) content. The hens in the experimental groups received a diet containing defatted HIM at levels of 5, 10, and 15% (HIM 5%, HIM 10%, and HIM 15%, respectively), at the expense of a proportionally decreasing content of SBM. During the 12-week experiment, the laying performance, biochemical and redox blood indices, and liver condition were examined. The cholesterol level, fatty acid profile, and malondialdehyde content in egg yolks were also evaluated to determine the dietary quality of the eggs. The inclusion of HIM at any level in the diet did not affect the laying performance parameters (*p* > 0.05). Increased serum Ca and uric acid contents were observed. There was no effect on the redox indices in plasma. The number of hepatocytes was decreased in the HIM-fed groups. The level of cholesterol in yolks was reduced, and the fatty acid profile showed significant changes. Despite the high lauric acid content in the *H. illucens* meal, it was present in trace amounts in yolks. In the HIM-fed groups, the levels of saturated fatty acids increased significantly, whereas those of unsaturated fatty acids decreased in the yolks in the same groups.

## 1. Introduction

According to the United Nations, egg production will increase to 89 million tons by 2030 [1], which will become a challenge to the feed industry to meet the nutritional requirements of laying hens, sustain high productivity, and maintain sustainable feed production. An alternative to soybean meal (SBM) in the feed industry is proteins of insect origin, which has recently attracted considerable and increasing interest as a feed material that provides higher local independence in the supply of feed protein with higher sustainability, as its production and use lead to lower carbon footprint and less negative impact on the environment and biodiversity compared with SBM. Moreover, consumers mostly have a positive attitude toward products from animals fed with insects [2], most likely because, for free-range hens, insects are a natural food source.

Following the European Union Commission Regulation 2017/893 of 24 May 2017, insects of some species, which are bred for the production of processed animal protein derived from insects, gained the status of “farmed animals”. Initially, the use of processed animal protein of insects was only possible in aquaculture and fur animals; however, the European Commission Regulation 2021/1372 of 17 August 2021 extended the scope of the target animals to include poultry and pigs.

Black Soldier Fly (*Hermetia illucens*) seems to be a promising species that can effectively convert organic matter from waste into insect biomass with a high protein and fat content, which can be further used in poultry nutrition [3]. The protein content of insects varies depending on the stage of development [4]. A higher total protein (Tp) content of 39.2% was observed in the larval stage, whereas in the black fly prepupae, its amount increased to about 40%. Using the appropriate production technology, insect meal can be obtained in which the protein level can be further increased by prior defatting of the insect. Such treatments result in 47.6–58.3% of protein in the meal [5], which is higher than those found in commercial SBM, ranging from 41.8 to 50% [6]. Moreover, *H. illucens* larvae from which the meal is produced have sufficient levels of lysine, methionine, threonine, isoleucine, and valine to meet the nutritional needs of poultry [7]. *H. illucens* as feed also contain antimicrobial peptides (AMPs), produced by their immune system, thus enabling them to thrive under harsh environmental conditions [8]. For instance, a new type of AMP—defensin-like peptide 4 —is identified in Black Soldier Fly, which displays antimicrobial activity against Gram-positive bacteria such as *Staphylococcus aureus*, *Staphylococcus epidermidis*, and *Bacillus subtilis* [9,10]. However, *H. illucens* larvae may have a negative impact on the health of laying hens or the nutritional value of eggs because they are rich in lipids, with saturated fatty acids (SFA) being predominant, especially medium-chain lauric, myristic, and palmitic acids [11,12], along with a low content of omega-3 polyunsaturated fatty acids (n-3). Previous studies using *H. illucens* meal (HIM) have shown that the complete substitution of SBM in hen diet may lead to reduced laying performance as evidenced by reduced feed intake and a decrease in average egg weight [13]. Thus, this study aimed to determine the possibility of partial graded replacement of SBM with defatted HIM in the diet of laying hens, without negative effects on performance indices, dietary egg quality, and health.

## 2. Materials and Methods

### 2.1. Animals, Experimental Design, and Diets

For this experiment, 96 17-week-old Bovans Brown laying hens were purchased from a commercial source and kept in enriched cages on a wire-mesh floor. Two birds were placed in each cage measuring 30 cm × 120 cm × 50 cm, corresponding to 3600 cm^2^ of total floor area. During the pre-experimental period, which lasted from 17 to 24 weeks of age, the hens were housed under standard climate-controlled conditions and fed a standard commercial diet. The experimental period lasted 12 weeks, from the 25th to the 36th week of age, in which the laying hens were housed under standard climatic conditions (19 ± 2 °C; 50–65% relative humidity; 14 L:10 D light program). The water and feed in mashed form were offered to all birds *ad libitum*. The birds were randomly divided into four groups, each containing an equal number of 12 replicate cages with two hens per cage. The hens from the control group received a feed mixture containing 20% of postextraction SBM, with no HIM content (HIM 0%). The birds in the experimental groups received a diet containing defatted *H. illucens* meal at a level of 5, 10, and 15% (HIM 5%, HIM 10%, and HIM 15%, respectively) at the expense of a proportionally decreasing content of postextraction SBM. All diets met the nutritional requirements for laying hens following Polish recommendations [14]. The nutritional composition of the experimental diets was determined based on the chemical composition of the raw feed materials, and their metabolizable energy values were calculated based on equations from the European Tables [15]. The nutritional values of *H. illucens* meal and SBM used in the experiment are presented in Table 1, and the composition and the nutritional value of the experimental diets are presented in Table 2. The fatty acid (FA) profile of the experimental diets, HIM and SBM, is presented in Table 3.

### 2.2. Sample Collection

During the 12-week experiment, feed intake, the number of eggs, and their weight were recorded, and the laying performance, the egg weight, the daily mass of eggs per hen, daily feed intake, and feed conversion per egg and per 1 kg of eggs were determined.

Two eggs from each cage were collected at the end of the 36th week of age. Yolks were separated and frozen at −20 °C until the FA composition and the cholesterol content of the yolks were determined. Additionally, during this period, two additional eggs from each cage were collected and stored for 14 days in a refrigerator (c.a. 5 °C); then, the yolks were separated, frozen, and lyophilized to determine the malondialdehyde content (MDA) using the thiobarbituric acid (TBA) method.

At the end of the experimental period, one hen from each replicate cage was randomly selected, weighed, and slaughtered by cervical dislocation after electrical stunning. Immediately after slaughter, blood was collected in tubes without an anticoagulant. The blood samples were then centrifuged at 3000× *g* for 10 min to obtain serum for further analysis of biochemical and oxidative status indices. Heart, gizzard, liver, pancreas, spleen, and abdominal fat samples were separated and weighed to determine their relative weight to live body weight. Liver samples were collected for histological examination, and the rest of the tissue was frozen for further analysis of the fat and MDA contents.

### 2.3. Analytical Procedure

In accordance with the guidelines of Commission Regulation (EC) No. 152/2009 of 27.01.2009, the dry matter (Annex III A), crude protein (Annex III C), crude ash (Annex III M), crude fat (Annex III H), crude fiber (Annex III I), and amino acid (Annex III F) contents, excluding tryptophan, were determined for the experimental diets, HIM and SBM. The tryptophan content was determined after alkaline hydrolysis according to procedure SOP M.006 issue 6 of 27 January 2020. Micronutrients and macronutrients in the feed materials and experimental diets were determined using flame atomic absorption according to SOP M.010 issue 4 of 24 February 2020, for copper, manganese, iron, and zinc and SOP M.008 issue 5 of ’24 February 2020, for calcium, magnesium, sodium, and potassium. The FA contents in HIM and SBM, as well as in the egg yolks, were determined using gas chromatography according to procedure P 015 issue 2 of 1 March 2016. The cholesterol content in the egg yolks was determined using the gas chromatography method according to SOP M.023 issue 2 of 27 January 2020. The MDA levels in the egg yolks and liver samples were determined using the TBA method according to Pikul et al. [16].

The following biochemical indices were determined in the blood serum samples using commercial kits (PZ Cormay Inc., Lomianki, Poland): Tp; albumin; triglycerides (TG); total cholesterol (TC); high-density lipoprotein; low-density lipoprotein; uric acid (UA); Ca, P, Mg, and Fe concentrations; and enzyme activities of alanine aminotransferase, aspartate aminotransferase, lactate dehydrogenase, alkaline phosphatase, amylase, and lipase.

The serum levels of superoxide dismutase (SOD), catalase (CAT), glutathione peroxidase (GPx), and MDA were measured using commercially available ELISA kits from the Bioassay Technology Laboratory (Shanghai, China), according to the manufacturer’s instructions.

**Figure 1:**
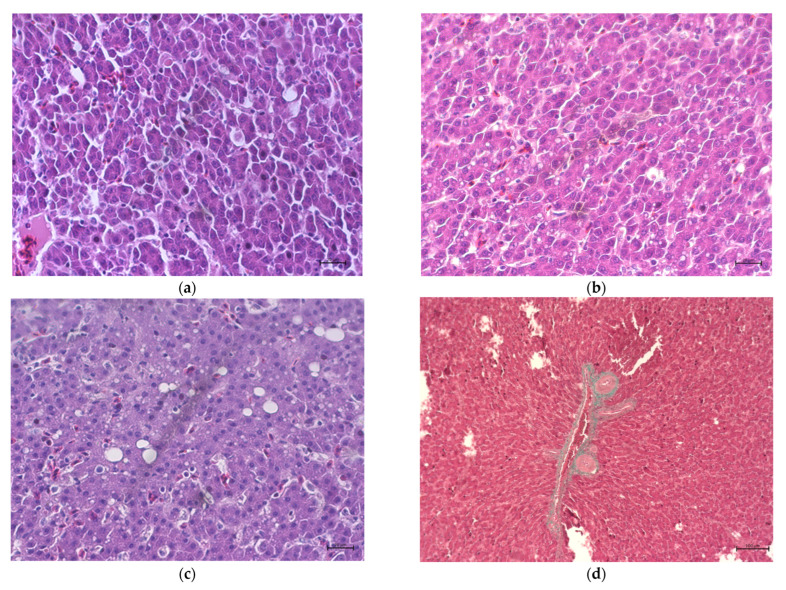
Representative photographs for histopathological changes in the liver of laying hens: (**a**) 1—no steatosis or single lipid droplets in the cells; (**b**) 2—visible lipid droplets in a large part of the cells (about 30–45%), morphologically altered cells; (**c**) 3—extensive steatosis, more than 50% of cells contain lipid droplets and/or fat vacuoles, cells morphologically altered, visible changes in the cytoplasm of cells, and nuclei often arranged peripherally; Hematoxylin and eosin staining. Magnification 40×; (**d**) collagen fiber imaging (Masson Goldner staining) of liver tissues. Magnification 10×.

For histological examinations, liver tissue samples were fixed in 4% buffered formaldehyde (pH 7.0) for 24 h, dehydrated in a graded series of ethanol, cleared with a nonpolar solvent, and then embedded in paraffin (Shandon Histoplast, Thermo Scientific, Cheshire, UK). Then, 4-µm-thick cross-sections were cut using a microtome (Thermo Scientific Microm HM 340 E, Walldorf, Germany) and then placed on a microscopic slide (Pathosolutions; ElektroMed, Niepolomice, Poland). The slides were stained with Harris hematoxylin and alcoholic eosin Y (ElektroMed, Niepolomice, Poland). The level of fibrosis was assessed using the Masson Goldner Trichrome Staining Kit (Diapath, Martinengo, Italy). The stained slides were observed using a light microscope (Axio Lab.A1 Carl Zeiss, Oberkochen, Germany) that was equipped with an Axiocam Color 105 camera (Carl Zeiss Microscopy, Oberkochen, Germany) using the graphical analysis software ZEN 2.3 blue edition software version 2.3 (Carl Zeiss Microscopy, Jena, Germany) and ImageJ version 1.53 (US National Institutes of Health, Bethesda, MD, USA). The number of hepatocytes per 0.1 mm^2^ of the cross-sectional area of the liver samples was counted. For each hen, two slides with five regions of interest (ROI) per slide were analyzed, and the hepatocytes present in each ROI were counted using a Cell counter plugin for ImageJ software. The average number of hepatocytes from all ROI per unit area was then calculated. The hepatic steatosis level was scored according to the 3-point scale (Figure 1):

1—No steatosis or single lipid droplets in the cells;

2—Visible lipid droplets in a large part of the cells (about 30–45%), morphologically altered cells;

3—Extensive steatosis, more than 50% of cells contain lipid droplets and/or fat vacuoles, cells morphologically altered, visible changes in the cytoplasm of cells, and nuclei often arranged peripherally.

### 2.4. Statistical Analysis

The data were analyzed using STATISTICA, version 13.3 (StatSoft Inc., Tulsa, OK, USA). For statistical analyses, each replicate cage was treated as an experimental unit. The Shapiro–Wilk test for normality of the data distribution and Levene’s test for homogeneity of variance were used. For normality and variance homogeneity, one-way ANOVA was carried out, followed by Duncan’s multiple-range post hoc test. For nonnormally distributed data, the Kruskal–Wallis test was carried out. When significant differences were detected using the Kruskal–Wallis test, a post hoc test for pairwise multiple comparisons of the ranked data was used. Values of *p* ≤ 0.05 were considered significant.

## 3. Results

The laying performance of the hens is presented in Table 4. Dietary HIM administration did not significantly affect the performance indices (*p* > 0.05). The mean laying rate, averaged across all dietary treatments throughout the entire experimental period (25–36 weeks of age), was 98.0%; daily egg mass, 57.7 g/hen; egg weight, 58.9 g; daily feed consumption, 120 g/hen; and feed conversion, 122.4 g of feed/egg or 2.08 g of feed/g of eggs.

The dietary HIM administration at all levels showed no significant differences (*p* > 0.05) in the relative weights of the gizzard, heart, liver, spleen, and pancreas, as well as in the relative abdominal fat weight (Table 5). Although the differences were not confirmed statistically, the increase in the relative weight of abdominal fat was observed by 21, 29.4, and 9.26% in HIM 5%, HIM 10%, and HIM 15% group, respectively, when compared with the control group.

The effects of dietary HIM administration on the biochemical indices are presented in Table 6. HIM did not significantly change most of the serum biochemical indices, except for the serum UA and Ca contents. The UA content of the HIM 5% and HIM 10% groups was significantly increased compared with that of the HIM 0% group. The UA content of the HIM 15% group was comparable to that of the HIM 0% and HIM 10% groups. All dietary HIM levels increased the serum concentration of Ca compared with the control group, whereas no significant differences between all HIM groups were observed.

Serum redox indices are presented in Table 7. The SOD, CAT, GPx, and MDA concentrations were not affected by any of the dietary HIM levels (*p* > 0.05).

The HIM administration in the diets of laying hens did not significantly affect the liver MDA and liver fat contents, as shown in Table 8 (*p* > 0.05). However, it increased the fat content, and the values for the HIM 5%, HIM 10%, and HIM 15% groups were 8.3, 9.4, and 7.7%, respectively, higher than that of the control group.

As presented in Table 9, the histological analysis did not indicate the negative effects of HIM administration on the mean score for liver steatosis as the accumulation of lipid droplets in the liver sections did not differ significantly between all groups (*p* > 0.05). Moreover, the liver tissue in all experimental groups was free of pathologic fibrosis. However, the number of hepatocytes significantly decreased in the HIM 10% and HIM 15% groups, compared with the control group. In some hens with high levels of steatosis cells (scored at three points), irregular structure and distribution of hepatocytes were observed. Furthermore, balloon cells near lipid droplets were also observed in these birds.

The results of the analysis of cholesterol level, FA profile, and MDA content in the egg yolks are summarized in Table 10. Significantly decreased TC levels were observed in all HIM groups, compared with the control group, with no differences between HIM groups (*p* = 0.020). In eggs collected at 36 weeks of age and refrigerated for 14 days, the MDA content of the yolks of all HIM groups was comparable to that of the control group; however, the MDA content of the yolks was significantly higher in the HIM 10% group than in the HIM 5% group.

In egg yolks, the lauric acid, myristic acid, and SFA contents were significantly increased in the HIM 10% and HIM 15% groups, proportionally to the increasing HIM content in the diet, compared with the values observed in the control group; however, the arachidic acid, docosahexaenoic acid, unsaturated fatty acids (UFA), polyunsaturated fatty acids (PUFA) n-3, and UFA/SFA contents were decreased. The total monosaturated fatty acids content was not affected. The linoleic acid and gamma-linolenic acid contents were decreased in the HIM 10% group, and the linolenic acid, total PUFA, and PUFA-6 contents were significantly decreased in the HIM 5% and HIM 10% groups than in the control group. As a consequence, with the increase in the content of HIM in the diet, the content of desirable fatty acids significantly decreased, whereas the PUFA 6/3 content significantly increased in the HIM 15% group, compared with the control group.

## 4. Discussion

In the present study, the partial replacement of SBM with defatted HIM up to the level of 15% in the diet of laying hens was not found to affect the laying rate. Similar results were reported in a study by Mwaniki et al. [17], where 10 or 15% of SBM was replaced with HIM with no effect on the laying rate, whereas 17% of insect meal as a total substitute of SBM resulted in a significant reduction in laying productivity [13]. In the present study, HIM administration did not affect the egg weight, whereas Mwaniki et al. [17] reported a linear decrease in egg mass. The possible reason for this effect is a lower supply of amino acids or a 35% lower supply in HIM diets, relative to the control group, of linoleic acid, which is known to contribute to egg size [18]. In the present study, the difference in the linoleic acid content in the FA profile between HIM 15% and the control diet was ca. 20%. Feed conversion ratio (FCR), which was expressed as the gram of feed per gram of produced eggs in the present experiment, remained unaffected, contrary to the results of Mwaniki et al. [17], Marono et al. [13], and All-Qazzaz et al. [19], who reported an increased FCR in groups fed with HIM, even at a level of 5% in the diet [19]. It is worth noting that some experiments took longer than ours, and with the long-term use of insect meal, there may be some changes in health and metabolic status that may affect the laying performance. Cutrignelli et al. [20], studying the effect of complete replacement of SBM with defatted HIM meal for 21 weeks, observed a strong reduction in protein digestibility (−13.6%), confirming that chitin, which is present in HIM as the primary component of the exoskeleton of insects, is a limiting factor in the digestibility of crude protein, which was also proven *in vitro* [21]. Furthermore, the digestibility of insect protein alone, depending on the species, is significantly lower compared with protein of plant origin and ranges from 45 to 66.9% [22]. Moreover, altered intestinal morphology, which was reflected by shorter villi, deeper crypts, and reduced villi/crypt ratio, was observed in laying hens [19] or broiler chickens [23] fed HIM diets, which may be another explanation for the reduced protein utilization.

In the present study, the HIM diets did not significantly affect the relative weights of organs, whereas an increase in the relative weights of abdominal fat was observed. Mwaniki et al. [17] reported an increased liver weight in hens fed with an HIM diet, with no effect on gizzard, small intestine, and pancreas weights. In the present study, although no statistical differences in relative liver weight or liver fat content were confirmed, increases in the liver fat content of 8.3, 9.4, and 7.7% were observed in the HIM 5%, HIM 10%, and HIM 15% groups, respectively. Histological examination indicated a significant decrease in the number of hepatocytes, with an increase in the proportion of HIM in the diet of the hens and a slight increase in the incidence of liver steatosis, although not statistically confirmed. However, this effect did not result in an increase in liver enzyme activities. In broiler chickens [23] or in Muscovy ducks [24], HIM administration did not affect liver histology. However, a longer experiment would answer whether long-term HIM feeding may be a predisposing factor for inducing fatty liver with metabolic disturbances of this key organ, since highly productive laying hens are prone to increased fat accumulation in this organ and frequent hemorrhage [25], which is associated with intensive yolk synthesis.

The experimental diets were calculated to provide an equal level of Ca; however, the analyzed content of Ca was 43.6, 43.1, 45.4, and 40.7 g/kg in the HIM 0%, HIM 5%, HIM 10%, and HIM 15% groups, respectively. Despite the lower Ca content of the HIM 15% diet, plasma calcium levels increased significantly in all groups of HIM-fed hens. This may be due to the prebiotic properties of chitin contained in the insect’s exoskeleton. By modulating the intestinal microflora, it leads to increased production of short-chain fatty acids (SCFAs) [26], increased acetic and butyric acid contents, and enhanced Ca absorption in the distal intestine [27]. Similar results were reported by Marono et al. [13], who reported increased circulating serum Ca levels in hens receiving 17% of HIM in the diet, despite a similar Ca concentration in the control and experimental diets.

In the present study, significant increases in the serum uric acid level were observed in the HIM 5% and HIM 10% groups. The higher UA content may be due to the difference in amino acid utilization [28]. Nevertheless, the recorded uric acid values were within the normal reference range as the other biochemical parameters tested [29]. An increased plasma uric acid content was also observed in broiler chickens receiving a diet with 30% black soldier larvae [30].

Similar to the present results of unaffected serum TG and TC levels, Bovera et al. [31], using a *Tenebrio molitor* meal as a total replacement for SBM in the diet, also observed no significant differences. However, Morono et al. [13] reported a reduced plasma TG and TC content in hens fed an HIM diet. Such an effect may be related to chitin, whose derivative chitosan prevents the complete emulsification of fats in the intestines, can bind fats, and thus prevent their absorption [32].

High animal productivity is often accompanied by oxidative stress, which indicates a reduction in the antioxidant capacity [33], i.e., detoxification [34]. No changes were observed in redox indices, such as SOD, CAT, GPx, and MDA, which is in line with the results of Kozlowski et al. [35] of MDA and CAT plasma levels in turkeys. However, Liu et al. [36] observed increased T-SOD and CAT activities and decreased MDA content with increasing HIM levels; however, the maximum HIM level in the diet in this study was 5%.

While considering the dietary quality of eggs, emphasis is placed on the lipids in the yolk, which constitute 65% of the dry matter of the yolk [37]. Significant differences were observed in the majority of the yolk FA contents, indicating that HIM modifies lipid metabolism. In the yolk lipids produced with HIM diets, a significantly higher SFA content was observed, especially in myristic and lauric acids, with lower proportions of PUFA and FA regarded as desirable, compared with the eggs of the control group. All these effects are regarded as negative from the consumers’ perspective. The most surprising observations concerned the lauric acid content (C12), which is the dominant FA of *H. illucens*, indicating that the insect meal was its major source in the experimental diets. With the increase in the level of HIM in the diet, the C12 content in the yolk increased, but despite a significant increase (*p* < 0.001), the analyzed content remained at a lower level, ranging from 0.0267 to 0.08 g per 100 g of the total FA content. Similar results were obtained by Heuel et al. [12], who reported that in insect-based diets, the net transfer from the diet to the egg yolk was less than 1% for lauric acid, whereas the net transfer for myristic and palmitic acids was approximately 30% and 100%, respectively. They provided two explanations for such a phenomenon. First, they suggested that SCFAs are used in other functions, such as energy metabolism, and are not necessarily deposited in the egg yolk. Second, chain elongation takes place through de novo lipogenesis (enterohepatic de novo lipogenesis). The results obtained for SFA in the present study are also consistent with those obtained by Secci et al. [1]. The positive effect observed in all groups receiving HIM diets was the decreased yolk cholesterol level. Zotte et al. [38] showed no difference in the cholesterol content in quail eggs. They suggest that the cholesterol level does not depend on the diet used but rather on intraorganismal transformations. However, it could be the result of chitin in the HIM diet, which was proven to lower the cholesterol level in eggs [39]. The degree of lipid oxidation in the egg yolk was examined using the TBA assay. The results obtained were inconsistent as a significant decrease in the MDA content was observed in the HIM 10% group compared with the HIM 5% group.

## 5. Conclusions

The inclusion of HIM in the diet for 12 weeks did not worsen the laying performance or affect the function of the liver and pancreas. HIM did not significantly alter most of the serum biochemical indices but increased the serum Ca content. Thus, it may be regarded as an efficient alternative protein source for laying hens. However, it negatively altered the liver histology and unfavorably changed the yolk FA profile, which needs further investigation.

## Figures and Tables

**Table 1:** Nutritive value of the *Hermetia illucens* meal and soybean meal.

Nutrient Composition [g/kg]	*H. illucens* Meal	Soybean Meal
Dry matter	943.0	913.6
Crude ash Crude fat	93.7 116.2	59.4 23.2
Crude protein	480.4	451.7
Crude fiber	92.6	36.1
Sodium Potassium Magnesium Calcium Zinc Manganese Copper Iron Phosphorus	1.43 16.9 4.26 16.9 0.147 0.205 0.0117 0.350 12.2	0.045 24.7 2.85 2.25 0.0441 0.0256 0.0128 0.0819 6.35
Aspartic acid	38.55	54.01
Threonine	17.29	18.78
Serine	18.94	23.71
Glutamic acid	47.30	79.56
Proline	26.02	20.33
Glycine	25.20	19.35
Alanine	35.89	21.12
Valine	25.65	21.30
Isoleucine	34.77	21.27
Leucine	31.39	32.88
Tyrosine	29.18	18.49
Phenylalanine	19.26	22.98
Histidine	13.27	12.07
Lysine	28.97	29.12
Arginine	21.28	34.18
Cysteine	3.59	6.24
Methionine	6.72	6.15
Tryptophan	5.33	7.04

**Table 2:** Composition and nutrient content of experimental diets.

Ingredient [g/kg]	HIM 0%	HIM 5%	HIM 10%	HIM 15%
*H. illucens* meal	0	50	100	150
Wheat	362.55	342.1	349.78	354.22
Maize	220	250	250	250
Soybean meal	200	150	100	50
Wheat bran	70	65	61	60
Rapeseed oil	35	32	30	28
Limestone	91.8	91.5	91	91
Monocalcium phosphate	11	9.3	7.8	6
Sodium chloride	3	3	3	3
DL-Methionine	1.5	1.32	1.15	0.98
L-Lysine hydrochloride	0.15	0.78	1.3	1.8
Vitamin–mineral premix *	5	5	5	5
Analyzed chemical composition (g/kg):				
Metabolizable energy (MJ/kg) ^1^	11.6	11.6	11.6	11.6
Crude protein	166.0	166.5	169.3	169.3
Calcium	43.6	43.1	45.4	40.7
Phosphorus	6.10	6.42	6.17	5.86
Aspartic acid	13.94	14.32	12.64	14.91
Threonine	5.2	5.87	5.34	6.42
Serine	7.7	8.43	7.84	9.68
Glutamic acid	34.18	33.63	29.76	34.14
Proline	9.4	10.82	10.02	13.09
Glycine	6.98	7.17	6.92	8.39
Alanine	7.84	8.51	9.04	11.01
Valine	7.35	7.57	7.66	8.92
Isoleucine	6.46	6.33	6.52	7.48
Leucine	12.77	12.25	12.07	13.32
Tyrosine	5.92	6.53	7.03	7.65
Phenylalanine	8.46	7.95	7.87	8.69
Histidine	4.53	4.53	4.54	5.06
Lysine	7.75	8.02	7.96	9.31
Arginine	10.48	9.6	8.94	9.16
Cysteine	2.64	2.61	2.53	2.59
Methionine	4.52	4.32	4.36	4.41
Tryptophan	2.32	2.39	2.44	2.61

* The premix provided per 1 kg of diet: vitamin A—10,000 IU (retinol); vitamin D3—2000 IU (cholecalciferol); vitamin E—20 IU (dl-alpha-tocopherol); vitamin K3—1.5 mg (menadione); vitamin B1—1 mg (thiamine); vitamin B2—4 mg (riboflavin); vitamin B6—1.5 mg (pyridoxine); vitamin B12—0.02 mg (cyanocobalamin); biotin—0.05 mg Ca-pantothenate—8.7 mg; niacin—20 mg; folic acid—0.8 mg; choline chloride—200 mg; manganese—85 mg; zinc—60 mg; iron—45 mg; copper—8 mg; iodine—1 mg; selenium—0.25 mg; ^1^ Calculated according to [15].

**Table 3:** Fatty acid profile in the experimental diets, soybean meal and *Hermetia illucens* meal (g/100 g of total fatty acids).

	Experimental Diets					
Items	HIM 0%	HIM 5%	HIM 10%	HIM 15%	HIM	SBM
C8—caprylic acid	0.00	0.00	0.00	0.00	0.00	0.00
C10—capric acid	0.00	0.15	0.27	0.40	1.61	0.00
C12—lauric acid	0.00	4.16	8.37	12.92	44.09	0.00
C14—myristic acid	0.08	1.25	2.19	2.95	9.48	0.15
C16—palmitic acid	10.31	10.67	10.77	11.12	14.81	17.55
C16-1—palmitoleic acid	0.22	0.59	0.89	1.11	3.19	0.35
C18—stearic acid	2.19	2.13	2.1	2.13	2.65	4.79
C18-1—oleic acid	50.14	45.53	42.81	39.66	14.77	14.46
C18-2—linoleic acid	28.74	27.55	25.03	23.05	7.7	47.27
Gamma18-3—gamma-linolenic acid	0.00	0.00	0.00	0.00	0.01	0.00
C20—arachidic acid	0.4	0.36	0.33	0.3	0.08	0.19
C18-3—linolenic acid	7.55	7.31	6.98	6.1	1.5	14.88
C20-4—arachidonic acid	0.00	0.00	0.00	0.00	0.03	0.00
C22-1—erucic acid	0.13	0.11	0.1	0.09	0.01	0.02
EPA—eicosapentaenoic acid	0.00	0.00	0.00	0.01	0.02	0.00
DHA—docosahexaenoic acid	0.00	0.00	0.00	0.00	0.01	0.00
SFA	13.21	18.92	24.19	29.98	72.77	23.02
UFA	86.79	81.08	75.81	70.02	27.23	76.98
MUFA	50.49	46.23	43.8	40.86	17.96	14.83
PUFA	36.3	34.86	32.01	29.16	9.28	62.15
PUFA-6	28.74	27.55	25.03	23.05	7.74	47.27
PUFA-3	7.56	7.31	6.98	6.1	1.54	14.88
DFA	88.98	83.22	77.91	72.15	29.88	81.77
UFA/SFA	6.57	4.29	3.13	2.34	0.37	3.34
MUFA/SFA	3.82	2.44	1.81	1.36	0.25	0.64
PUFA/SFA	2.75	1.84	1.32	0.97	0.13	2.7
PUFA 6/3	3.8	3.77	3.58	3.78	5.04	3.18

HIM—*Hermetia illucens* meal; SBM—soybean meal; SFA—saturated fatty acids; UFA—unsaturated fatty acids; MUFA—monosaturated fatty acids; PUFA—polyunsaturated fatty acids; PUFA-6: C18-2, C20-4, gamma18-3; PUFA-3: C18-3, EPA, DHA; DFA—desirable fatty acids: UFA + C18.

**Table 4:** Effects of dietary treatments on laying performance from 25 to 36 weeks of age hens (*n* = 12).

Items	HIM 0%	HIM 5%	HIM 10%	HIM 15%	SEM	*p*-Value
Laying rate [%]	98.5	97.4	97.2	98.9	0.284	0.085
Daily mass of eggs [g per hen]	57.5	58.1	57.6	57.6	0.288	0.89
Egg weight [g]	58.4	59.7	59.3	58.2	0.278	0.197
Daily feed intake [g per hen]	122	117	121	119	1.11	0.190
FCR per one egg [g]	124	120	125	121	1.14	0.127
FCR per g of egg [g]	2.12	2.02	2.11	2.07	0.0232	0.159

*p* > 0.05; HIM—*Hermetia illucens* meal; FCR—feed conversion ratio; SEM—standard error of mean.

**Table 5:** Effects of dietary treatments on the relative weights of organs and abdominal fat in laying hens at the 36th week of age.

Items	HIM 0%	HIM 5%	HIM 10%	HIM 15%	SEM	*p*-Value
Gizzard [%]	1.15	1.13	1.12	1.07	0.022	0.688
Liver [%]	1.32	1.34	1.28	1.30	0.024	0.844
Pancreas [%]	0.17	0.16	0.17	0.16	0.004	0.545
Spleen [%]	0.082	0.076	0.071	0.083	0.002	0.084
Heart [%]	0.31	0.31	0.29	0.32	0.004	0.250
Abdominal fat [%]	3.67	4.44	4.75	4.01	0.175	0.137

*p* > 0.05; HIM—*Hermetia illucens* meal; SEM—standard error of mean.

**Table 6:** Effects of dietary treatments on the biochemical indices of hens’ blood collected at the 36th week of age (*n* = 12).

Items	HIM 0%	HIM 5%	HIM 10%	HIM 15%	SEM	*p*-Value
Tp [g/dL]	5.22	5.4	5.64	5.5	0.101	0.329
Alb [g/dL]	2.36	2.46	2.46	2.44	0.027	0.516
TG [mg/dL]	1123	1264	1318	1276	29.6	0.103
TC [mg/dL]	129.9	153.4	161.9	151.9	5.63	0.156
HDL [mg/dL]	46.6	54.8	55	56.8	1.46	0.058
LDL [mg/dL]	44.2	43	43.7	45.6	1.17	0.881
UA [mg/dL]	3.99 ^c^	5.79 ^a^	5.47 ^ab^	4.68 ^bc^	0.185	0.001
Alt [U/L]	2.91	4.53	3.6	4.35	0.325	0.205
Ast [U/L]	225	198	199	207	4.19	0.069
LDH [U/L]	1775	1692	1831	1512	74.9	0.258
Alp [U/L]	578	462	601	632	32.1	0.203
Amylase [U/L]	379	390	374	334	11.4	0.332
Lipase [U/L]	9.21	9.52	9.53	9.23	0.075	0.262
Ca [mg/dL]	25.0 ^b^	27.2 ^a^	27.4 ^a^	26.9 ^a^	0.319	0.028
P [mg/dL]	5.14	5.92	6.12	5.75	0.148	0.080
Mg [mg/dL]	3.41	3.77	3.71	3.66	0.061	0.182
Fe [mcg/dL]	566	579	599	631	12.4	0.278

^a, b, c^—mean values within the same row followed by different superscripts differ significantly at *p* ≤ 0.05; HIM—*Hermetia illucens* meal; Tp—total protein; Alb—albumin; TG—triglycerides; TC—total cholesterol; HDL—high-density lipoprotein; LDL—low-density lipoprotein; UA—uric acid; Alt—alanine aminotransferase; Ast—aspartate aminotransferase; LDH—lactate dehydrogenase; Alp—alkaline phosphatase; Ca—calcium; P—phosphorus; Mg—magnesium; Fe—iron; SEM—standard error of mean.

**Table 7:** Redox status indices of laying hens’ blood collected at the 36th week of age.

Items	HIM 0%	HIM 5%	HIM 10%	HIM 15%	SEM	*p*-Value
SOD [ng/mL]	15.4	14.9	13.3	16.4	17.0	0.759
CAT [ng/mL]	78.4	59.0	50.5	68.3	5.32	0.286
GPx [ng/mL]	10.8	8.68	7.12	10.5	0.64	0.136
MDA [nmol/mL]	47.2	39.1	33.3	38.4	2.70	0.520

*p* > 0.05; HIM—*Hermetia illucens* meal; SOD—superoxide dismutase; CAT—catalase; GPx—glutathione peroxidase; MDA—malondialdehyde; SEM—standard error of mean.

**Table 8:** The malondialdehyde and fat contents and the number of hepatocytes in the liver tissue of laying hens at the 36th week of age.

Items	HIM 0%	HIM 5%	HIM 10%	HIM 15%	SEM	*p*-Value
MDA [mg/kg liver]	1.05	1.03	0.98	1.3	0.026	0.981
Fat content [%]	18.1	19.6	19.8	19.5	0.380	0.340

*p* > 0.05; HIM—*Hermetia illucens* meal; MDA—malondialdehyde; SEM—standard error of mean.

**Table 9:** The histological examination of the liver tissue of laying hens at the 36th week of age.

Items	HIM 0%	HIM 5%	HIM 10%	HIM 15%	SEM	*p*-Value
Liver steatosis scoring [points]	1.50	1.625	2.00	1.375	0.114	0.320
Liver fibrosis	nd	nd	nd	nd		
Number of hepatocytes [H/0.1 mm^2^]	2199 ^a^	2014 ^ab^	1841 ^b^	1872 ^b^	44.1	0.009

^a, b^—mean values within the same row followed by different superscripts differ significantly at *p* ≤ 0.05; HIM—*Hermetia illucens* meal; nd—not detected; H—number of hepatocytes; SEM—standard error of mean.

**Table 10:** The cholesterol and malondialdehyde contents and fatty acid profile of egg yolks collected at the 36th week of age.

Items	HIM 0%	HIM 5%	HIM 10%	HIM 15%	SEM	*p*-Value
Total cholesterol [mg/g yolk]	13.5 ^a^	12.7 ^b^	12.8 ^b^	12.5 ^b^	0.115	0.020
MDA [g/kg yolk]	0.65 ^ab^	0.66 ^a^	0.57 ^b^	0.62 ^ab^	0.012	0.036
Fatty acid [g/100 g of total fatty acids]						
C12—lauric acid	0.00417 ^c^	0.0267 ^bc^	0.0483 ^ab^	0.08 ^a^	0.0044	0.000
C14—myristic acid	0.357 ^c^	0.558 ^bc^	0.723 ^ab^	1.03 ^a^	0.0377	0.000
C16—palmitic acid	24.4	25.0	25.2	25.1	0.113	0.058
C16-1—palmitoleic acid	3.63	4.01	3.90	4.08	0.073	0.145
C18—stearic acid	8.49	8.47	8.68	8.55	0.080	0.796
C18-1—oleic acid	46.7	46.7	46.8	45.4	0.218	0.150
C18-2—linoleic acid	11.8 ^a^	11.0 ^ab^	10.7 ^b^	11.7 ^a^	0.156	0.028
Gamma18-3—gamma-linolenic acid	0.0658 ^a^	0.0533 ^ab^	0.0500 ^b^	0.0542 ^ab^	0.001	0.007
C20—arachidic acid	0.0492 ^a^	0.0450 ^ab^	0.0325 ^bc^	0.0283 ^c^	0.0017	0.000
C18-3—linolenic acid	0.710 ^a^	0.608 ^b^	0.588 ^b^	0.67 ^ab^	0.0153	0.012
C20-4—arachidonic acid	2.23 ^a^	2.07 ^b^	1.97 ^bc^	1.89 ^c^	0.0266	0.000
C22-1—erucic acid	0.0142 ^a^	0.0050 ^b^	0 ^b^	0 ^b^	0.0010	0.000
EPA—eicosapentaenoic acid	0.0100	0.0100	0.00917	0.0108	0.0003	0.271
DHA—docosahexaenoic acid	1.53 ^a^	1.46 ^ab^	1.36 ^bc^	1.33 ^bc^	0.0213	0.000
SFA	33.3 ^b^	34.1 ^ab^	34.7 ^a^	34.8 ^a^	0.128	0.000
UFA	66.7 ^a^	65.9 ^ab^	65.3 ^b^	65.2 ^b^	0.128	0.000
MUFA	50.3	50.7	50.7	49.5	0.188	0.110
PUFA	16.3 ^a^	15.2 ^b^	14.6 ^b^	15.6 ^ab^	0.192	0.013
PUFA-6	14.0 ^a^	13.1 ^bc^	12.7 ^c^	13.6 ^ab^	0.166	0.016
PUFA-3	2.25 ^a^	2.08 ^ab^	1.95 ^b^	2.01 ^b^	0.031	0.004
DFA	75.2 ^a^	74.4 ^b^	74.0 ^bc^	73.7 ^c^	0.134	0.000
UFA/SFA	2.00 ^a^	1.94 ^ab^	1.89 ^b^	1.87 ^b^	0.011	0.000
PUFA 6/3	6.25 ^b^	6.35 ^b^	6.5 ^b^	6.76 ^a^	0.052	0.002

^a, b, c^—mean values within the same row followed by different superscripts differ significantly at *p* ≤ 0.05; HIM—*Hermetia illucens* meal; MDA—malondialdehyde; SFA—saturated fatty acids; UFA—unsaturated fatty acids; MUFA—monosaturated fatty acids; PUFA—polyunsaturated fatty acids; PUFA-6: C18-2, C20-4, gamma18-3; PUFA-3: C18-3, EPA, DHA; DFA—desirable fatty acids: UFA + C18; SEM—standard error of mean.

## Data Availability

The data presented in this study are available within the article.
